# Supplementary material for: Spatial Transcriptomics Identifies Cellular and Molecular Characteristics of Scleroderma Skin Lesions: Pilot Study in Juvenile Scleroderma
Source: Int J Mol Sci. 2024 Aug 23;25(17):9182. doi: 10.3390/ijms25179182 (PMC11395166; doi:10.3390/ijms25179182)
Supplement: Supplementary file 1 [file ijms-25-09182-s001.zip › Supplementary Figures.pdf]

Spatial Transcriptomics Identifies Cellular and Molecular Characteristics of Scleroderma Skin Lesions: Pilot in Juvenile Scleroderma

Tianhao Liu [1,2], Deren Esencan [1,3], Claudia M. Salgado [1,4], Chongyue Zhao [1], Ying-Ju Lai [5], Theresa Hutchins [1,3], Anwesha Sanyal [1,3], Wei Chen [1,5,*] and Kathryn S. Torok [1,3,*]

[1] Department of Pediatrics, University of Pittsburgh School of Medicine, UPMC Children's Hospital of Pittsburgh, 4401 Penn Ave, Pittsburgh, PA 15224, USA; tianhao@pitt.edu (T.L.); esencand2@upmc.edu (D.E.); claudia.salgado@miami.edu (C.M.S.); chz113@pitt.edu (C.Z.); hutchinstr@upmc.edu (T.H.); sanyala@upmc.edu (A.S.)

[2] School of Medicine, Tsinghua University, Beijing 100084, China

[3] UPMC Scleroderma Center, University of Pittsburgh, Pittsburgh, PA 15224, USA

[4] UMMG Department of Pathology, Miller School of Medicine, Medical Campus, University of Miami, 1550 NW 10th Ave #118, Miami, FL 33136, USA

[5] Department of Biostatistics, University of Pittsburgh, Pittsburgh, PA 15224, USA; yil346@pitt.edu

* Correspondence: wei.chen@pitt.edu (W.C.); kathryn.torok@chp.edu (K.S.T.)

Supplemental Figures

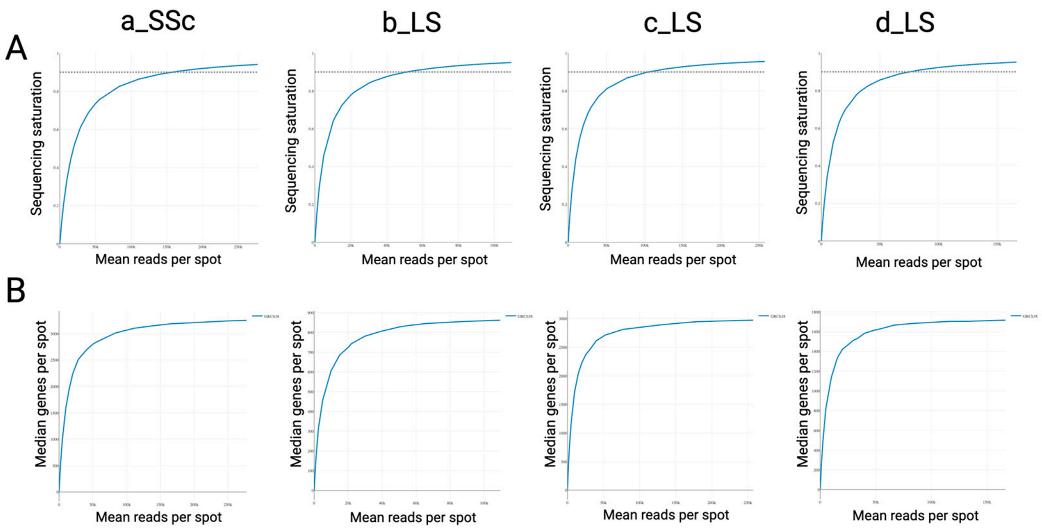

Supplementary Figure S1.

Saturation of sequencing. A. X axis is the mean reads per spot. Y axis is the saturation of sequencing. This indicates that saturation is high enough and increasing the number of sequenced reads cannot increase much saturation. B. X axis is mean reads per spot. Y axis is median genes per spot. This indicates that saturation is high enough and increasing the number of sequenced reads cannot increase the number of detected genes. Sample names: a_SSc sample a with systemic scleroderma, b_LS, c_LS, d_LS, sample b,c,d with localized

scleroderma.

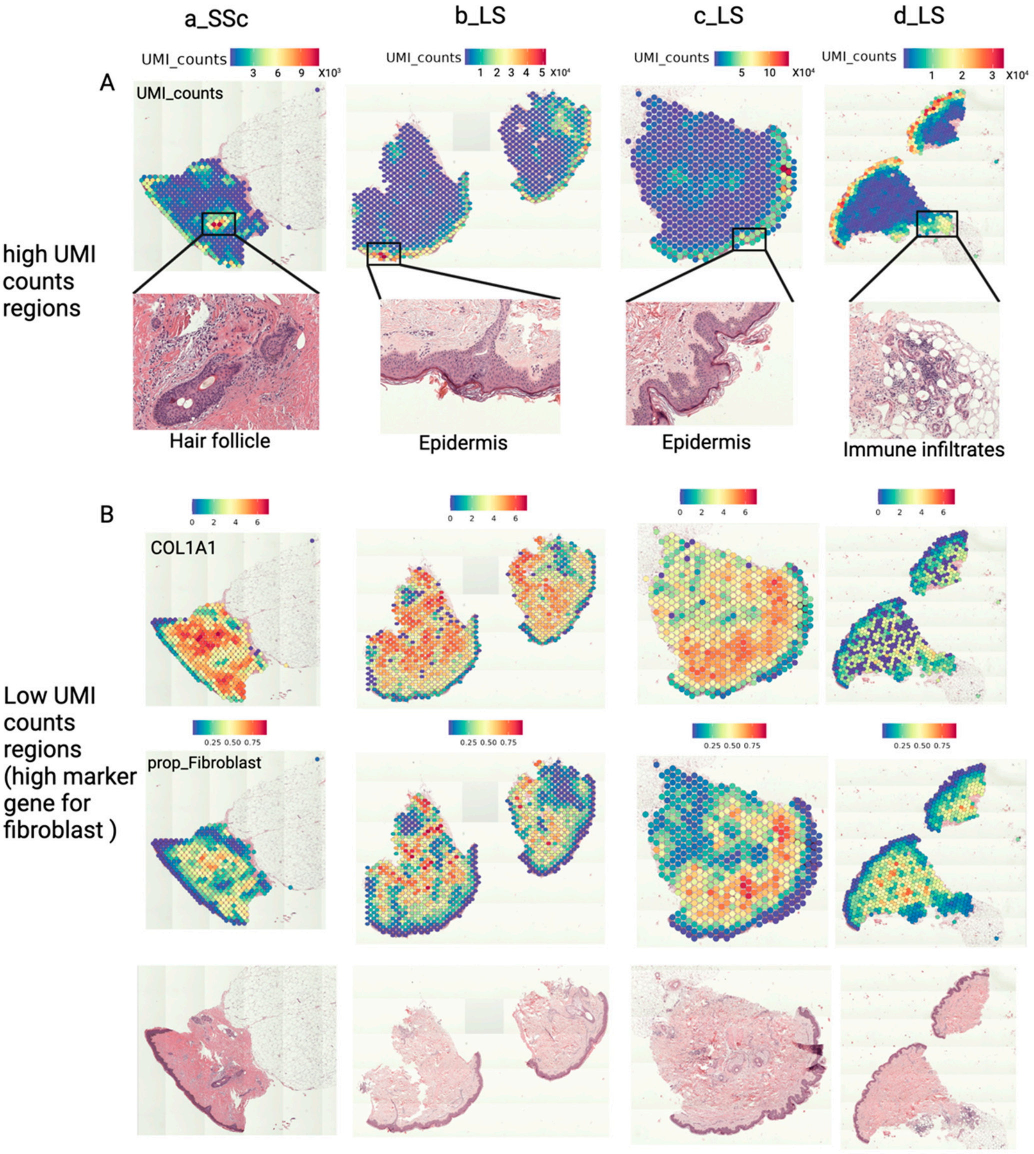

Supplementary Figure S2. Unique molecular index (UMI) count varies between different spatial regions. A. The UMI counts in hair follicle, epidermis and immune infiltrates is high. The density of nuclei is also high in these regions. B. The gene expression (COL1A1) and proportion of fibroblasts is high in collagen-rich areas with low UMI counts.

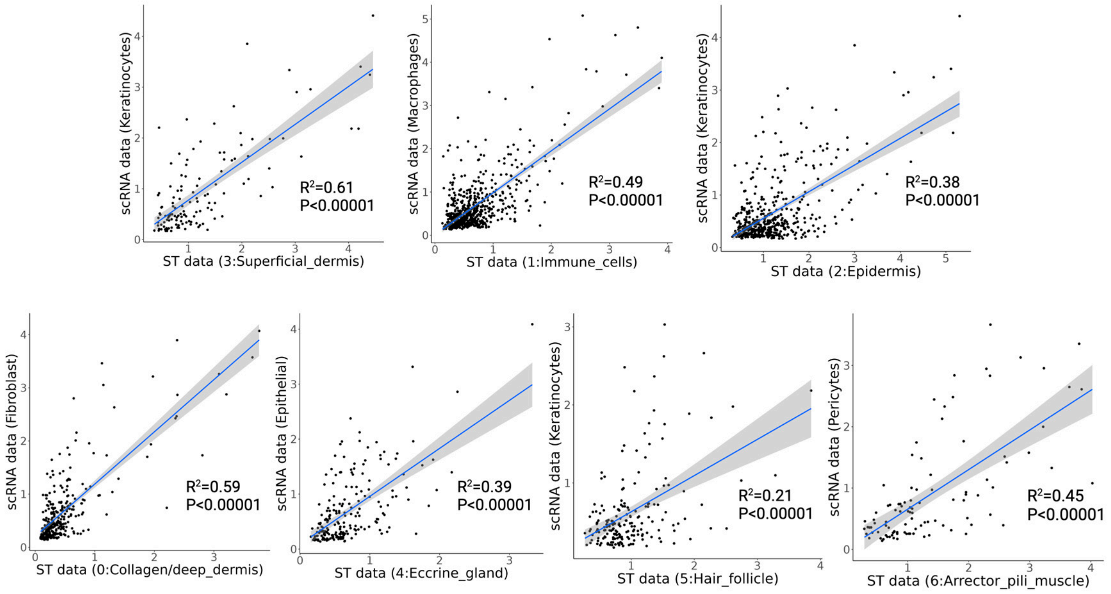

Supplementary Figure S3. Correlation between the gene expression in scRNA data and ST data. Each spot in the scatter plot indicates one gene. The X axis is averaged expression of this gene among spots in the indicated spatial domain (named in X axis). The Y axis is an average expression of this gene among cells that belong to the cell type harvesting the most overlapped marker genes with the spatial domain in X axis. For each scatter plot, only the overlapped marker genes between the spatial domain and scRNA cluster are plotted.

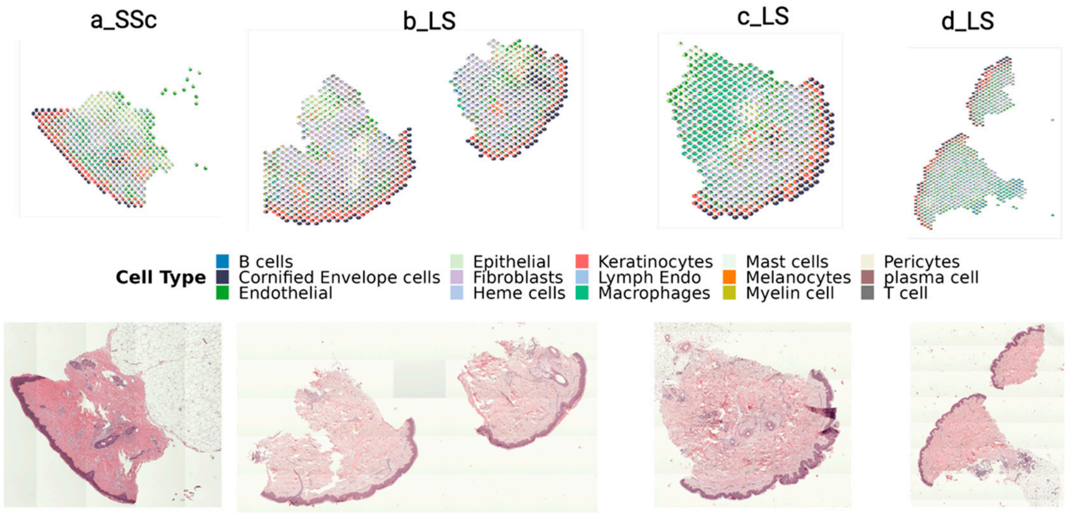

Supplementary Figure S4. Pie plots shows the proportion of each cell type in each spot. a_SSc, b_LS, c_LS and d_LS are four samples. The epidermis regions have high proportion of keratinocytes and cornified envelop cells.

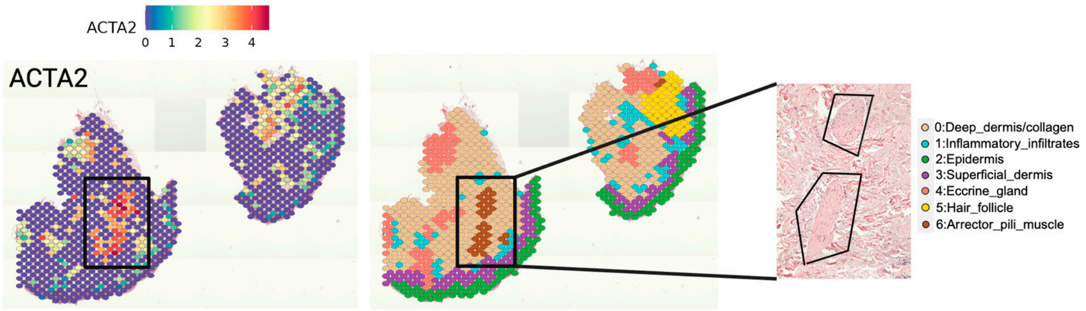

Supplementary Figure S5. The arrector pili muscle regions can be identified from gene expression. Expression of marker gene for Arrector pili muscle (ACTA2) *(left)*. The spatial domains detected with gene expression data *(middle)*. The H&E image of corresponding area *(right)*.

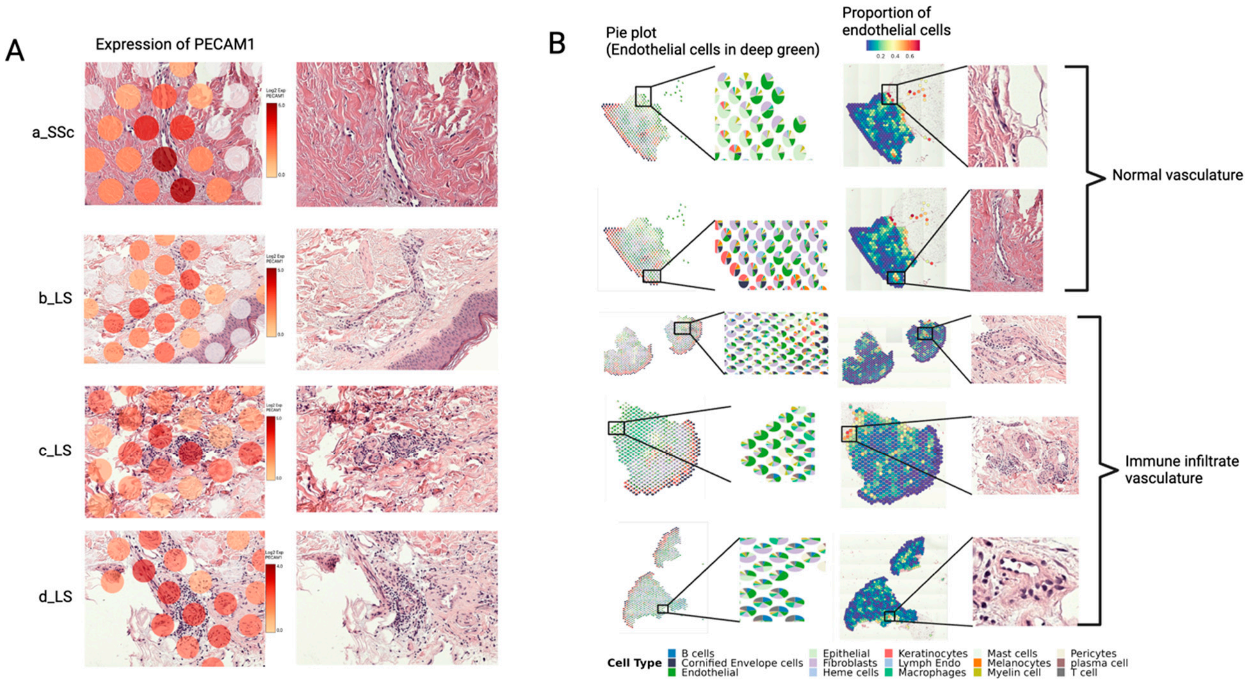

Supplementary Figure S6. Expression of marker gene PECAM1 and proportion of endothelial cells can distinguish blood vessels. (A) The area with high expression of PECAM1 covers area of blood vessel distinguished from H&E image. The gene expression image of first column was downloaded from Loupe Browser 8.0.0. (B) First two columns, the zoom in of pie plots in areas with high proportion of endothelial cells. The proportion of endothelial cells was shown in deep green. The third column, the proportion of endothelial cells was shown as the density of color. The fourth column, zoom in on area with high proportion of endothelial cells on the H&E image.

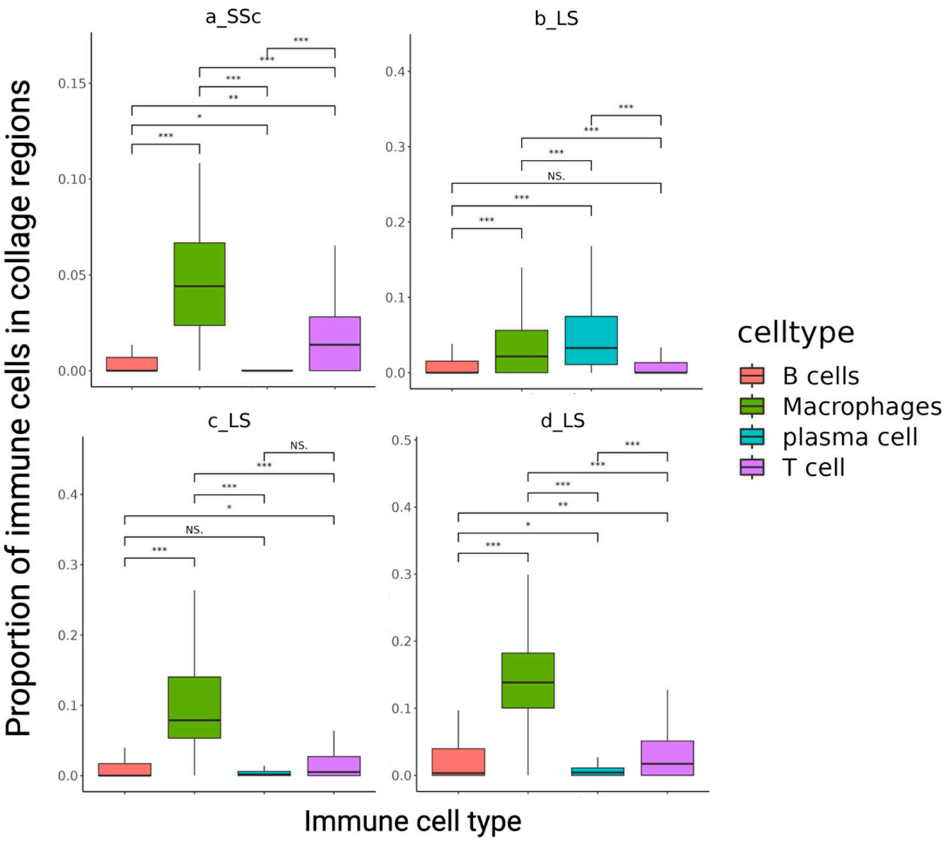

Supplementary Figure S7. Proportion of macrophages in collagen-rich area is higher than proportion of other immune cells. Four plots correspond to four different samples (a_SSc, b_LS, c_LS, d_LS). Y axis is the proportion of different types of immune cells (B cells, Macrophages, Plasma cells and T cell). The proportion of macrophage in cluster 0: Deep dermis/Collagen is higher than the proportion of other immune cells in sample a_SSc, c_LS and d_LS.

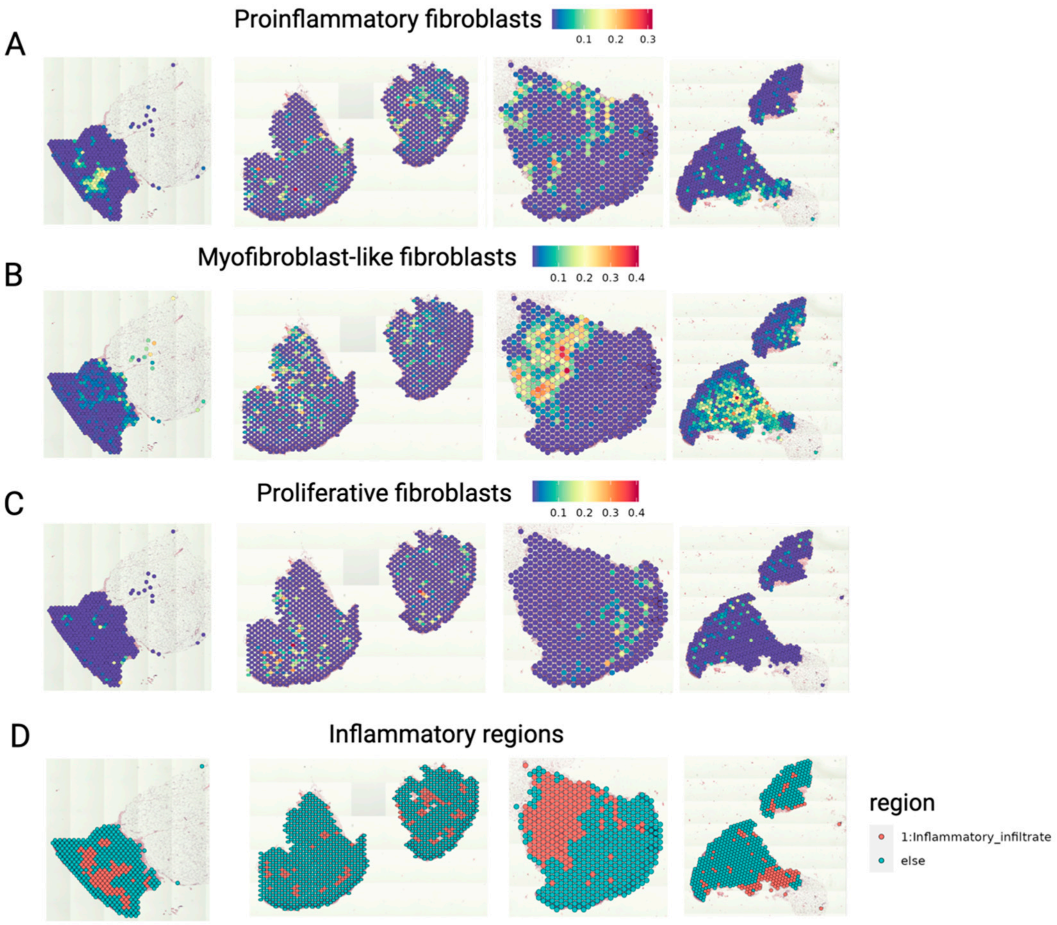

Supplementary Figure S8. Spatial distribution of three subtypes of fibroblasts. (A-C) Proportion of proinflammatory, myofibroblasts-like and proliferative fibroblasts. The proportion was calculated with cell type deconvolution method RCTD (robust cell type decomposition). (D) Region of inflammatory infiltrates in red. Other non-inflammatory regions in blue.
